# Unveiling protist diversity associated with the Pacific oyster *Crassostrea gigas* using blocking and excluding primers

**DOI:** 10.1186/s12866-020-01860-1

**Published:** 2020-07-03

**Authors:** Camille Clerissi, Laure Guillou, Jean-Michel Escoubas, Eve Toulza

**Affiliations:** 1 IHPE, Univ. Montpellier, CNRS, Ifremer, Univ. Perpignan Via Domitia, Perpignan, France; 2 PSL Université Paris: EPHE-UPVD-CNRS, USR 3278 CRIOBE, Université de Perpignan, 52 Avenue Paul Alduy, 66860 Perpignan Cedex, France; 3 Sorbonne Université, CNRS, UMR7144 Adaptation et Diversité en Milieu Marin, Ecology of Marine Plankton (ECOMAP), Station Biologique de Roscoff SBR, Roscoff, France; 4 IHPE, Univ. Montpellier, CNRS, Ifremer, Univ. Perpignan Via Domitia, Montpellier, France

**Keywords:** Holobiont, Metabarcoding, Microbiome, Ostreoida, *Crassostrea*

## Abstract

**Background:**

Microbiome of macroorganisms might directly or indirectly influence host development and homeostasis. Many studies focused on the diversity and distribution of prokaryotes within these assemblages, but the eukaryotic microbial compartment remains underexplored so far.

**Results:**

To tackle this issue, we compared blocking and excluding primers to analyze microeukaryotic communities associated with *Crassostrea gigas* oysters. High-throughput sequencing of 18S rRNA genes variable loops revealed that excluding primers performed better by not amplifying oyster DNA, whereas the blocking primer did not totally prevent host contaminations. However, blocking and excluding primers showed similar pattern of alpha and beta diversities when protist communities were sequenced using metabarcoding. Alveolata, Stramenopiles and Archaeplastida were the main protist phyla associated with oysters. In particular, *Codonellopsis*, *Cyclotella*, *Gymnodinium*, *Polarella*, *Trichodina*, and *Woloszynskia* were the dominant genera. The potential pathogen *Alexandrium* was also found in high abundances within some samples.

**Conclusions:**

Our study revealed the main protist taxa within oysters as well as the occurrence of potential oyster pathogens. These new primer sets are promising tools to better understand oyster homeostasis and disease development, such as the Pacific Oyster Mortality Syndrome (POMS) targeting juveniles.

## Background

The farmed oyster *Crassostrea gigas* is affected by the Pacific Oyster Mortality Syndrome (POMS) targeting juveniles [1, 2]. This disease is multifactorial due to abiotic factors (water temperature, seawater quality) [3], and biotic factors (development stage, interactions with microorganisms) [4, 5]. Among biotic factors, some are microbial pathogens (*Ostreid herpesvirus* OsHV-1 μVar, *Vibrio* spp.), but other microbes might play a positive role [6]. Indeed, oysters are associated with various microorganisms (called microbiota), and recently resistance to disease was linked to characteristics of the prokaryotic compartment [4].

Most analyses of oyster microbiota corresponded to prokayotes, but very little is known concerning microbial eukaryotes (hereafter named protists). This lack of knowledge is mainly explained by methodological issues. Indeed, while the 16S rRNA gene is succesfully used to characterize prokaryotic assemblages in metabarcoding surveys, the related rRNA marker gene for eukaryotes (18S) mostly amplified the abundant host DNA rather than associated protists. However, protists might play a role in oyster homeostasis. For example, *C. gigas* oysters are infected by several protistan parasites [7–11]. Among them, *Marteilia* and *Pseudoperkinsus* genera have negative impacts on the mollusc aquaculture production [12, 13]. Moreover, other protists such as *Alexandrium minutum* are detrimental for oysters [14], but also for human health [15].

A universal non-metazoan (UNonMet) primer set was developed in 2004 [16], but the expected product size (~ 600 bp) exceeded the size limit of MiSeq Illumina technology (2 × 300 bp, requiring overlap between read pairs). Recently, a study highlighted high performances of this primer set using in silico analyses, and proposed to use nested PCR (i.e., two-step PCR that consists in amplifying a shorter amplicon after a first PCR using the UNonMet primers) to tackle the amplicon size issue [17]. Although nested PCR might bias the abundance of environmental sequences, the comparison of UNonMet with universal primer sets (non-nested PCR) revealed however similar protist assemblages in this study.

Instead of developing and/or using primer sets that directly exclude the amplification of metazoans, another strategy would use a combination of a universal primer set and a blocking primer that specifically targets a region of the universal reverse primer. Blocking primers are modified at the 3′-end with a Spacer C3 CPG (3 hydrocarbons), thus the elongation is prevented during PCR and the targeted sequences are not amplified. Such an approach has the advantage of being very specific (excluding only sequences similar to the blocking primer), and has proven to be effective in the study of fish and krill gut contents [18, 19], coral-associated protists [20], and in the removal of metazoa sequences from seawater community samples [21].

As a consequence, in order to reveal protist diversity associated with *C. gigas* oysters, we developed a blocking primer associated with a commonly used primer set targeting the V4 loop of the 18S rRNA gene [22–28]. First, we computed in silico analyses to compare the blocking primer to the UNonMet primer set, but also to a primer set predicted to exclude most *C. gigas* sequences [20]. Secondly, we performed metabarcoding sequencing using a heterogeneous dataset of oyster samples (multiple origins and transplantations). This in vivo comparison was not done using the UNonMet primer set, because of issues related to large amplicon sizes when this study was done. This study aims at comparing different primer sets to describe protist diversity within *C. gigas* microbiota, and to discuss advantages and disadvantages of these different types of primers.

## Results

### In silico specificity of blocking and excluding primers

In order to describe protist diversity associated with oysters, a preliminary sequencing test was performed for a sample of *C. gigas* using a commonly used primer set targeting the V4 region of the 18S rRNA gene (Table 1). Because primers for 18SV4 were designed previously to amplify all eukaryotes [22], these sequencing tests showed as expected an excess of amplicons from *C. gigas*, representing ~ 99.7% of sequences (for a total of 2696 cleaned sequences).

**Table 1:** Blocking and excluding primers used for metabarcoding analyses

Marker region	Target	Forward (5′- > 3′)	Reverse (5′- > 3′)	Blocking primer (5′- > 3′)
18SV4	Eukaryota	CCAGCASCYGCGGTAATTCC	ACTTTCGTTCTTGATYRA	TCTTGACTAATGAAAACATGCTTGG
18SV1V2	Non-metazoa	ACCTGGTTGATCCTGCCA	GTARKCCWMTAYMYTACC	No blocking primer

**Fig. 1:**
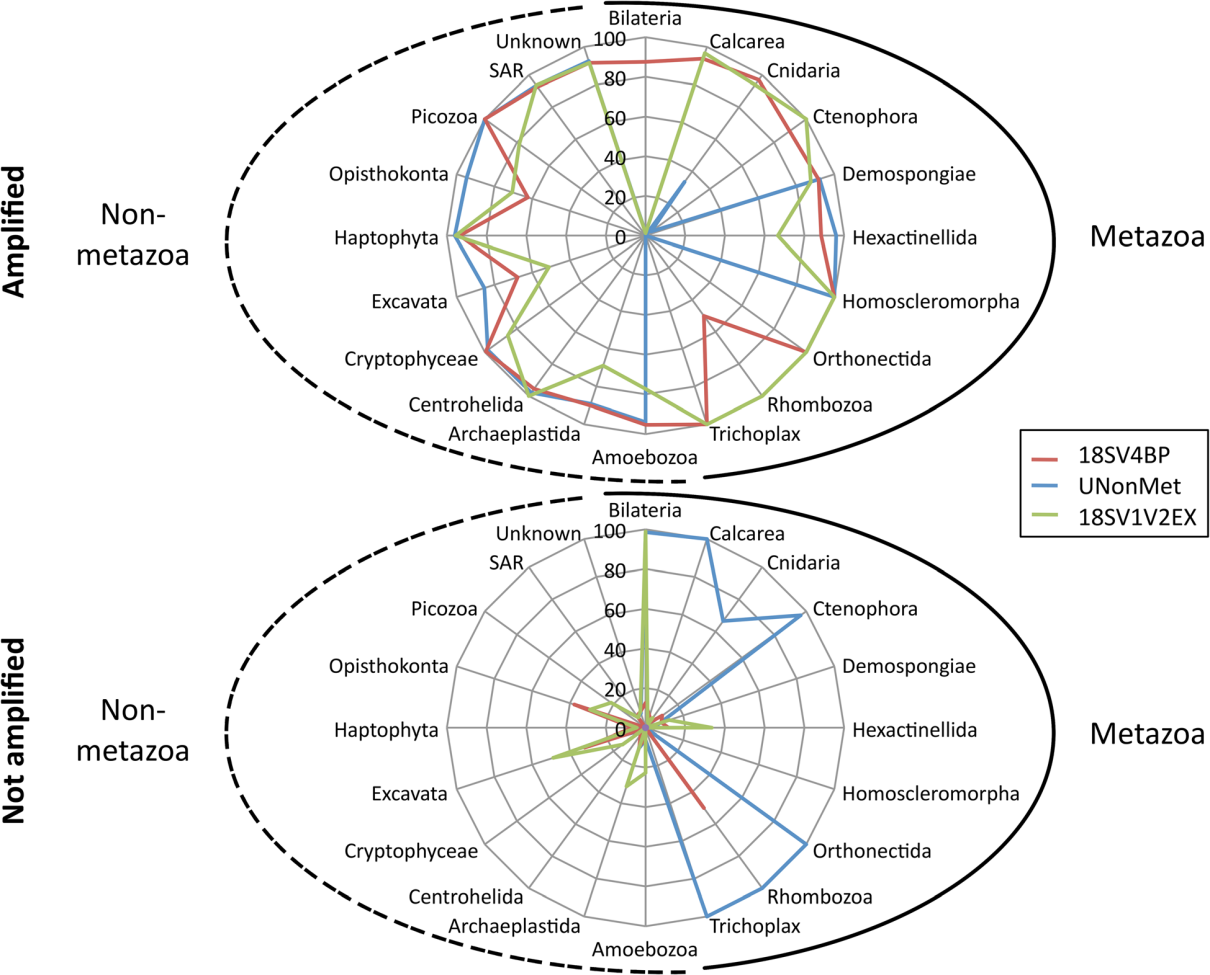
In silico specificity of blocking and excluding primers

Thus we designed blocking primers that specifically target the *Crassostrea* genus (Table 1) using the Silva SSU database (see Methods for more details). Briefly, to estimate the specificity of this blocking primer, we identified sequences of the Silva database that matched with both the primer set and the blocking primer. Only Ostreoida (oyster order) sequences from four genera (*Crassostrea*, *Hyotissa*, *Ostrea*, and *Saccostrea*) were removed by the blocking primer. Although not all (82%) Ostreoida sequences matched with the blocking primer, we found a very high in silico specificity for the *Crassostrea* genus (100%). In addition, this blocking primer did not remove protists found in the Silva database. Furthermore, we estimated the specificity of this blocking primer in association with the 18SV4 primer set (Table 1) against metazoan and non-metazoans sequences from the SSU Silva database. The term 18SV4BP was used hereafter for sequences obtained using the blocking primer targeting 18SV4. While the blocking primer excluded four Ostreoida genera, biases were identified for the 18SV4 primer set, particularly for Excavata and Opisthokonta (Fig. 1).

Then we compared 18SV4BP to two already published excluding primer sets: the UNonMet primers (also targeting the V4 loop of the 18S rRNA gene) [16], and a primer set (18SV1V2EX) that targeted the variable loops V1 and V2 of the 18S rRNA gene, predicted to prevent amplification of *C. gigas* (according to sequence comparison between primers and *C. gigas*) [20]. For metazoan sequences, these analyses highlighted that (i) UNonMet performed well to exclude most metazoan sequences (except for Cnidaria, Demospongiae, Hexactinellida, and Homoscleromporpha), and (ii) 18SV1V2EX mostly excluded Bilateria. For non-metazoan sequences, 18SV4BP performed better than 18SV1V2EX, but UNonMet tended to amplify a higher diversity than the two other primers.

To conclude, in silico analyses of blocking and excluding primers revealed that UNonMet was powerful to describe oyster-associated protists. Moreover, the blocking primer 18SV4BP was highly specific and removed only oyster sequences. While 18SV1V2EX excluded *Crassostrea* sequences, they might also exclude some protist groups (Amoebozoa, Archaeplastida, Cryptophyceae, Excavata, Opisthokonta, and Picozoa).

### Metabarcoding analyses of protist assemblages using biparental oysters families

**Fig. 2:**
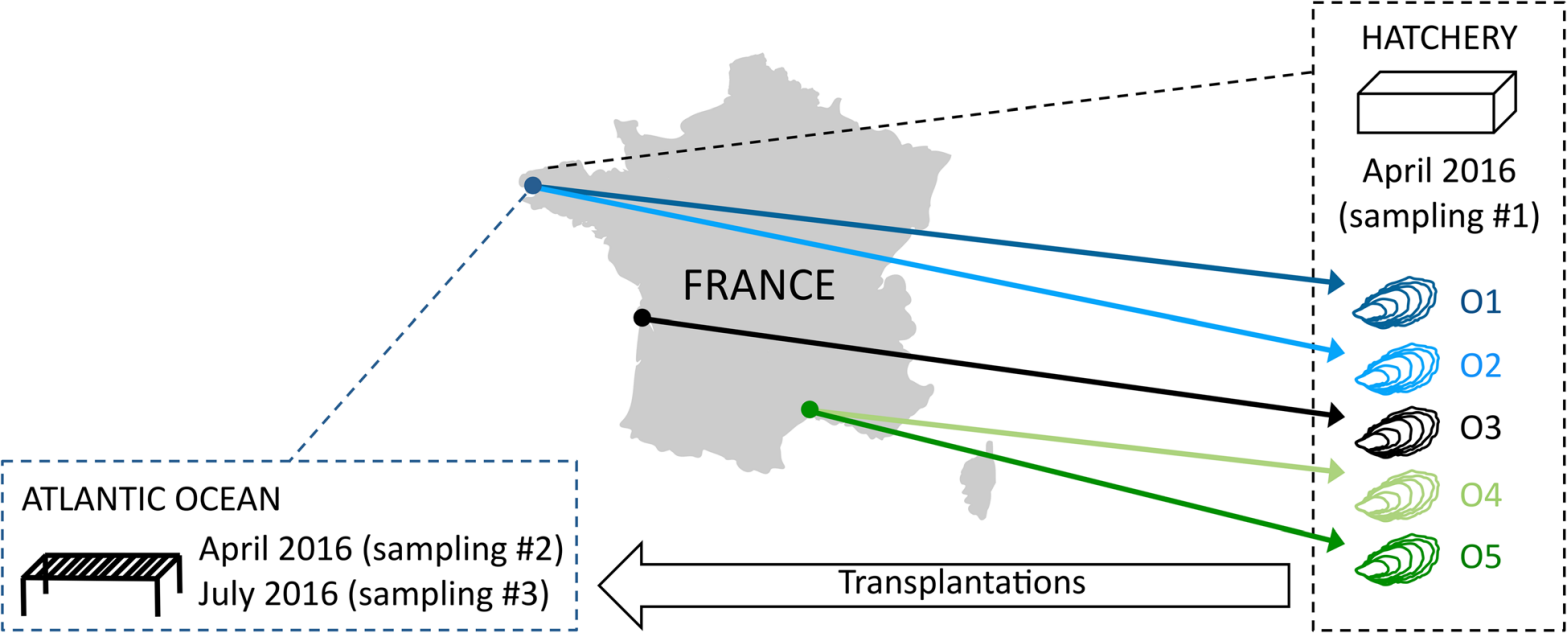
Oyster samples. Five biparental oyster families produced with broodstocks from different origins in terms of geography (Atlantic Ocean or Mediterranean Sea): O1, Logonna Daoulas (latitude: 48.335263; longitude: -4.317922); O2, Dellec (latitude: 48.353970; longitude: -4.566123); O3, Charente Maritime (latitude: 45.781741; longitude: -1.121910); O4, Vidourle (latitude: 43.553906; longitude: 4.095175); O5, pond of Thau (latitude: 43.418736; longitude: 3.622620). Oyster families were produced in hatchery, and placed for five days in natural environment in the Atlantic Ocean (latitude: 48.335263; longitude: 4.317922) at two time periods (April or July 2016). The map was modified from Wikimedia Commons (https://commons.wikimedia.org/wiki/File:France_all_regions.svg?uselang=fr), published under a Creative Commons license

Although the UNonMet excluding primers performed well in the in silico analyses, the amplicon size (~ 600 bp) was still a limitation for amplicon sequencing using MiSeq when this study was done. As a consequence, we only performed in vivo comparisons between 18SV4BP and 18SV1V2EX. Five biparental oyster families (O1-O5) of *C. gigas* were used in this study (Fig. 2 and Additional file 1: Table S1). They were produced within a hatchery (Argenton, France) using genitors from different origins (Atlantic Ocean or Mediterranean Sea). Protist assemblages were sampled from the first oyster generation kept in the hatchery (sampling #1), or placed in the environment at two different time periods (sampling #2 and #3). This dataset was thus heterogeneous and represented a high diversity of oyster-associated protists in order to compare blocking and excluding primer sets.

**Fig. 3:**
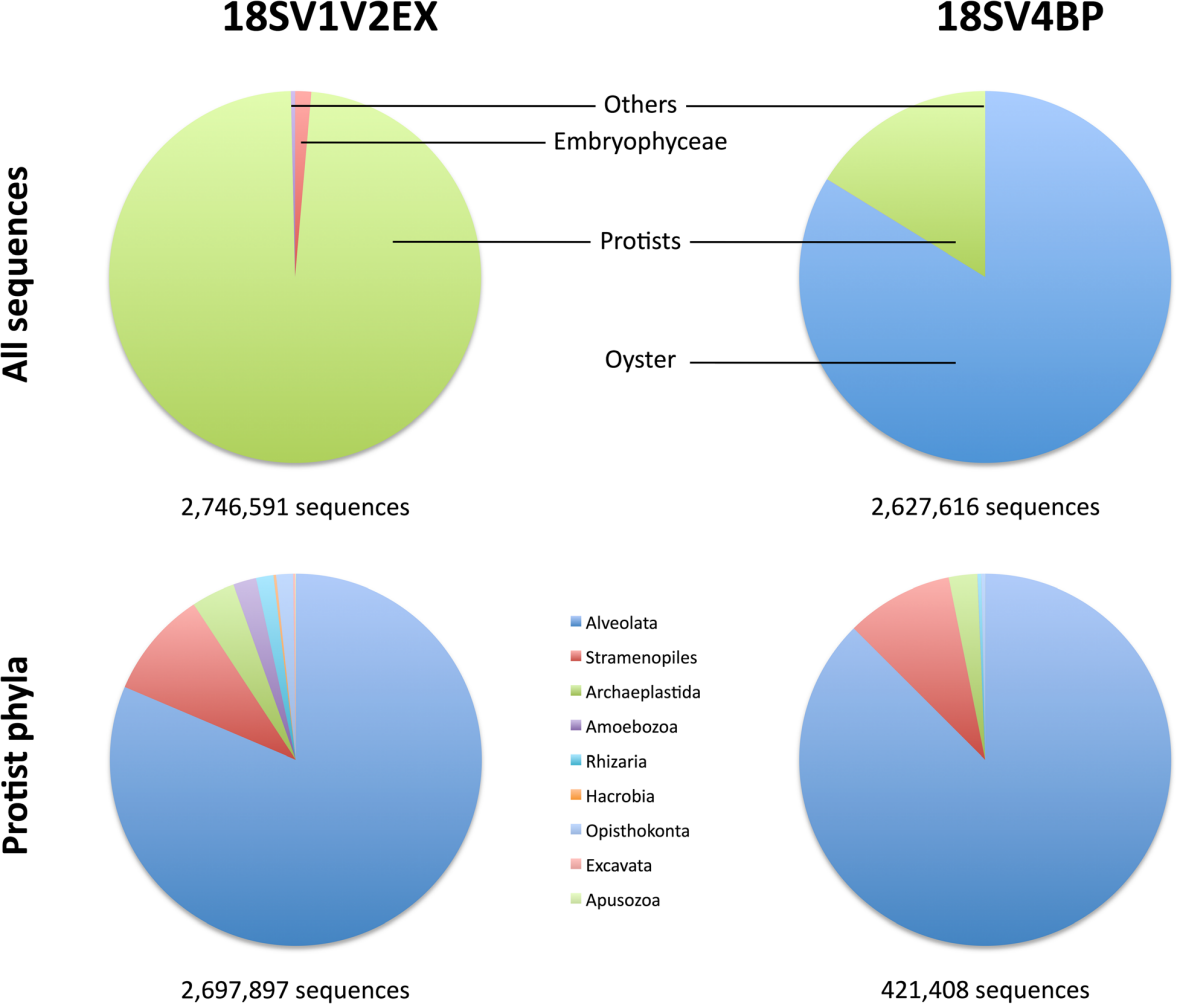
Sequences of high taxonomic ranks (oysters, Embryophyceae, protists) and protist phyla within oyster microbiota

First, we compared both marker regions for the abundances of total sequences at high taxonomic ranks: oyster, Embryophyceae, protists, and others (i.e., other metazoa and multi-affiliation). Similar numbers of sequences were obtained using 18SV1V2EX and 18SV4BP (Table 2). However, while host DNA was missing from 18SV1V2EX dataset, the use of 18SV4BP still displayed host contaminations (Fig. 3, Additional file 2: Fig. S1 and Additional file 3: Table S2). Accordingly, protist fractions were significantly higher for 18SV1V2EX than 18SV4BP (Table 2).

**Table 2:** Comparison of the abundances of total sequences and high taxonomic groups between 18SV1V2EX and 18SV4BP. Numbers into brackets are *p*-values. NS: not significant. 18SV1V2EX and 18SV4BP indicate which marker had significantly higher values

Taxa	18SV1V2EX vs. 18SV4BP
Total sequences	NS (*P* = 0.211)
Oyster	18SV4BP (*P* < 0.001)
Embryophyceae	18SV1V2EX (*P* < 0.001)
Protists	18SV1V2EX (*P* < 0.001)
Others	18SV4BP (*P* < 0.001)

### Nucleotide and alpha diversities of protist sequences

In order to perform rigorous comparisons of protist sequences between 18SV1V2EX and 18SV4BP, we decided to keep only samples having more than 5160 protist sequences. Moreover, the dataset was rarefied to this minimal value. While the whole 18SV1V2EX samples had more than 5160 protist sequences, only 34 out of 54 18SV4BP samples were kept for subsequent analyses (Additional files 4, 5, 6: Fig. S2, Tables S3-S4).

**Fig. 4:**
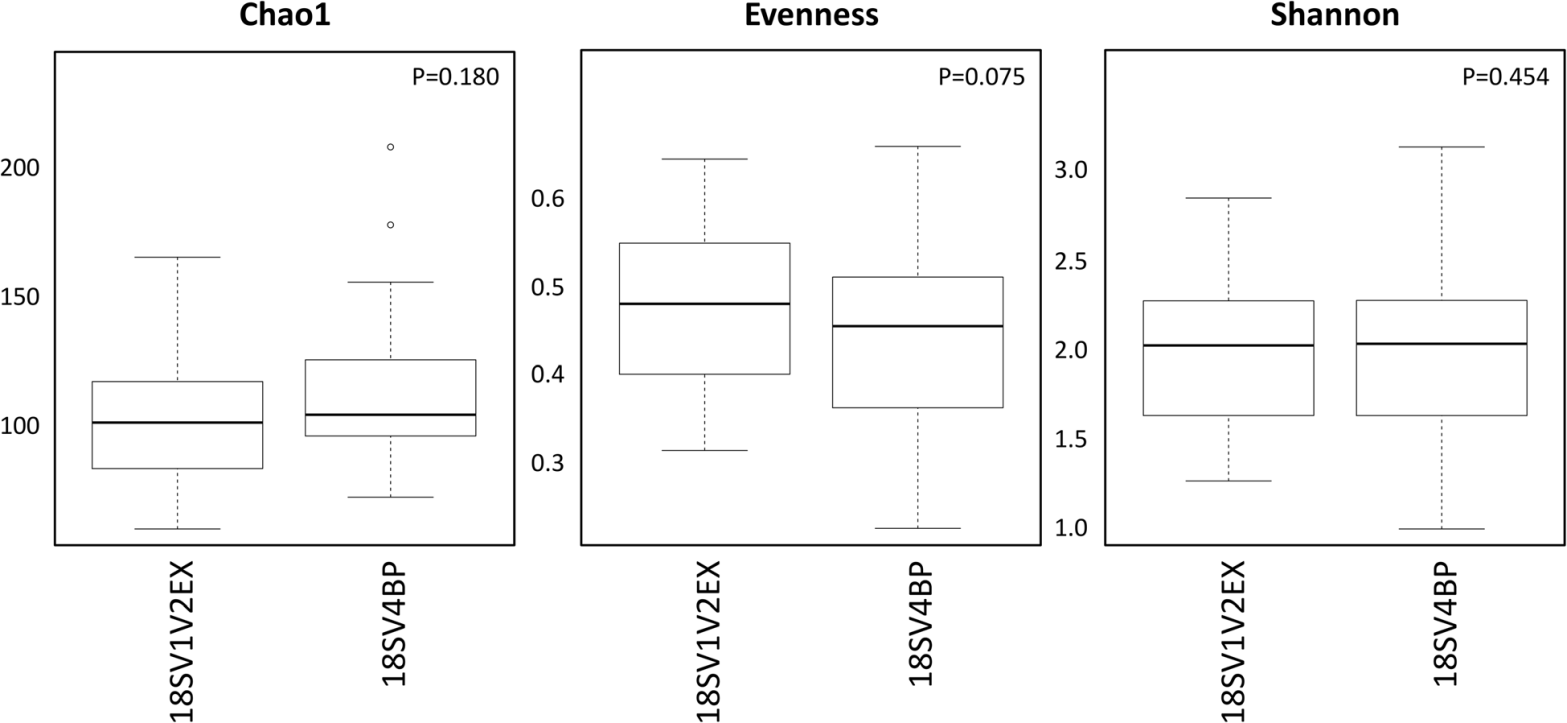
Comparison of alpha diversity indices for protists between 18SV1V2EX and 18SV4BP

The analysis of protist sequences showed that 18SV4BP amplicons (377 bp) were longer than 18SV1V2EX (304 bp) in average (Additional file 7: Fig. S3), and that nucleotide diversity of 18SV1V2EX (0.152) was higher than 18SV4BP (0.090). It suggested that although amplicons of 18SV1V2EX were shorter than 18SV4BP, the obtained diversity was higher for 18SV1V2EX. Because the same samples were analyzed using 18SV1V2EX or 18SV4BP, these observations might be the result of either a better resolution of the V1 and V2 loops of 18S rRNA gene or amplification of a more diverse set of protists. Furthermore, we compared both 18SV1V2EX and 18SV4BP for different alpha diversity indices (Additional file 8: Table S5), but no significant differences were found between both markers (Fig. 4).

### Composition of protist communities within oyster microbiota

**Fig. 5:**
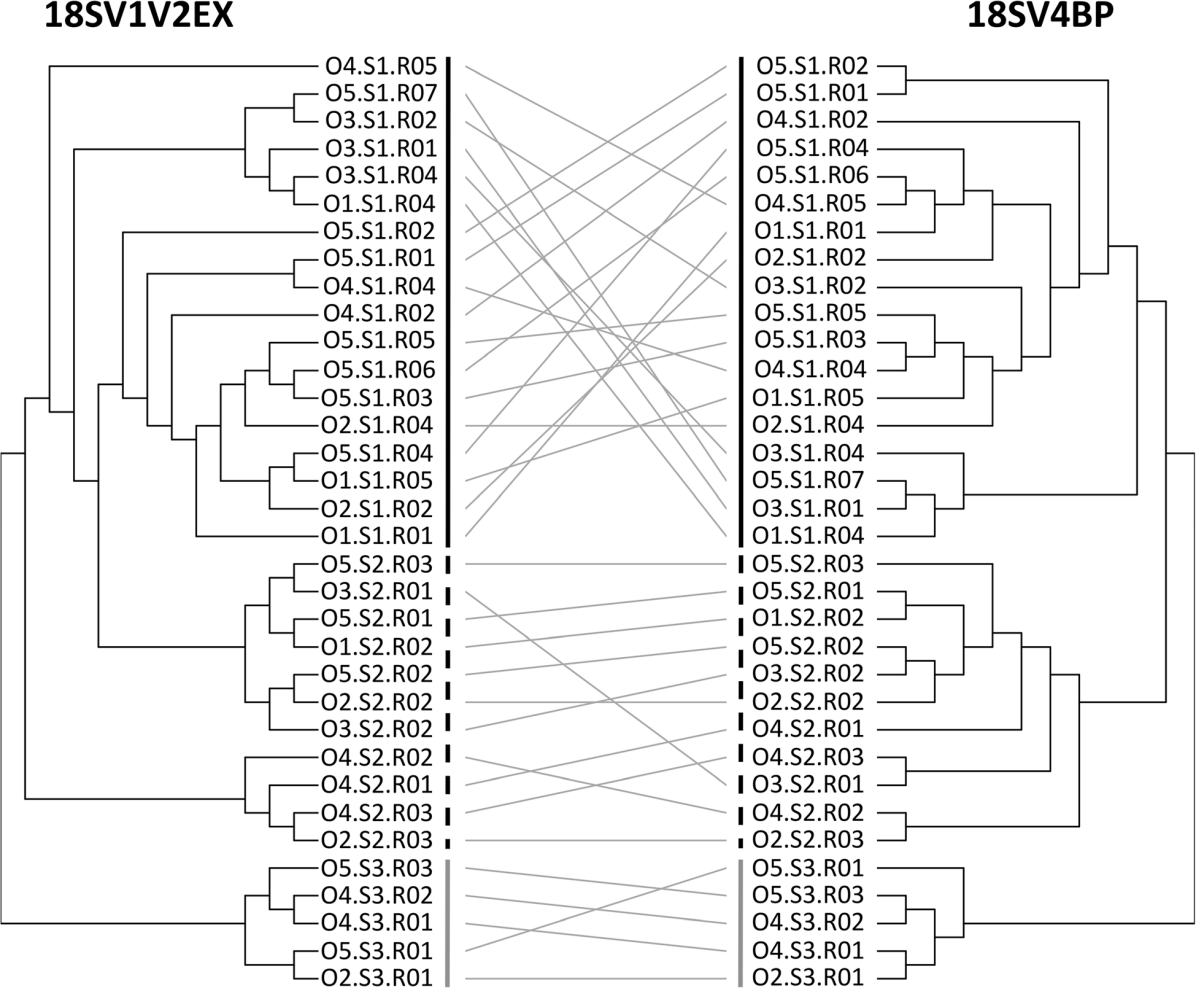
Clustering of microbial communities using 18SV1V2EX and 18SV4BP. Clusterings were computed using Bray-Curtis dissimilarities based on abundances of OTUs, and the average linkage method. O1-O5: five oyster families. Black, dashed and grey vertical lines indicate sampling #1, #2, and #3, respectively

**Fig. 6:**
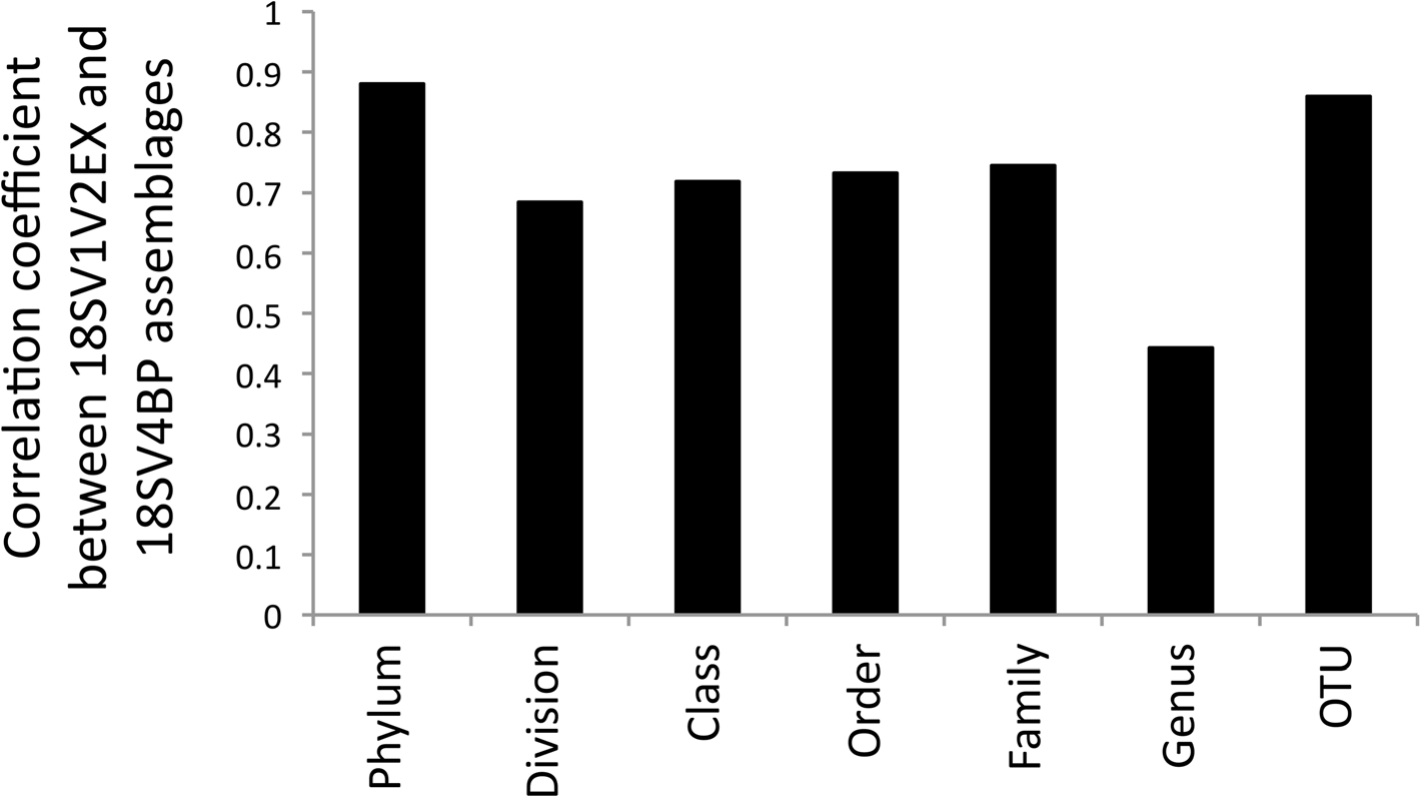
Comparison of protist community dissimilarities (Bray-Curtis) between 18SV1V2EX and 18SV4BP at different taxonomic ranks

First, we used the rarefied dataset and clustering analyses to study protist assemblages at the OTU scale. Both 18SV1V2EX and 18SV4BP showed similar patterns for protist assemblages (Fig. 5), and Bray-Curtis dissimilarities were significantly correlated (*r* = 0.86, *p* = 0.001, Mantel test). In particular, we found that protist assemblages were significantly linked to environmental conditions (S1-S3) (*p* = 0.001) rather than oyster families (O1-O5) (*p* > 0.4) for both 18SV4BP and 18SV1V2EX using PERMANOVA. Mantel tests were then computed at each taxonomic rank (from genus to phylum), and suggested that 18SV1V2EX and 18SV4BP mostly gave similar results (Fig. 6). For example, this study revealed that the main protist phyla were Alveolata, Stramenopiles and Archaeplastida (Fig. 3). Nevertheless, the lower correlation between 18SV1V2EX and 18SV4BP was obtained at the genus level (*r* = 0.44, *p* = 0.001, Mantel test). Surprisingly, both marker regions identified different dominant genus both within Alveolata (Additional files 5-6: Tables S3-S4). Indeed, *Codonellopsis* was the most abundant genus for 18SV1V2EX (10.30% and undetected for 18SV1V2EX and 18SV4BP, respectively), whereas *Woloszynskia* dominated protist assemblages using 18SV4BP (0.22 and 46.92% for 18SV1V2EX and 18SV4BP, respectively).

### Dominant genera within protist assemblages

**Fig. 7:**
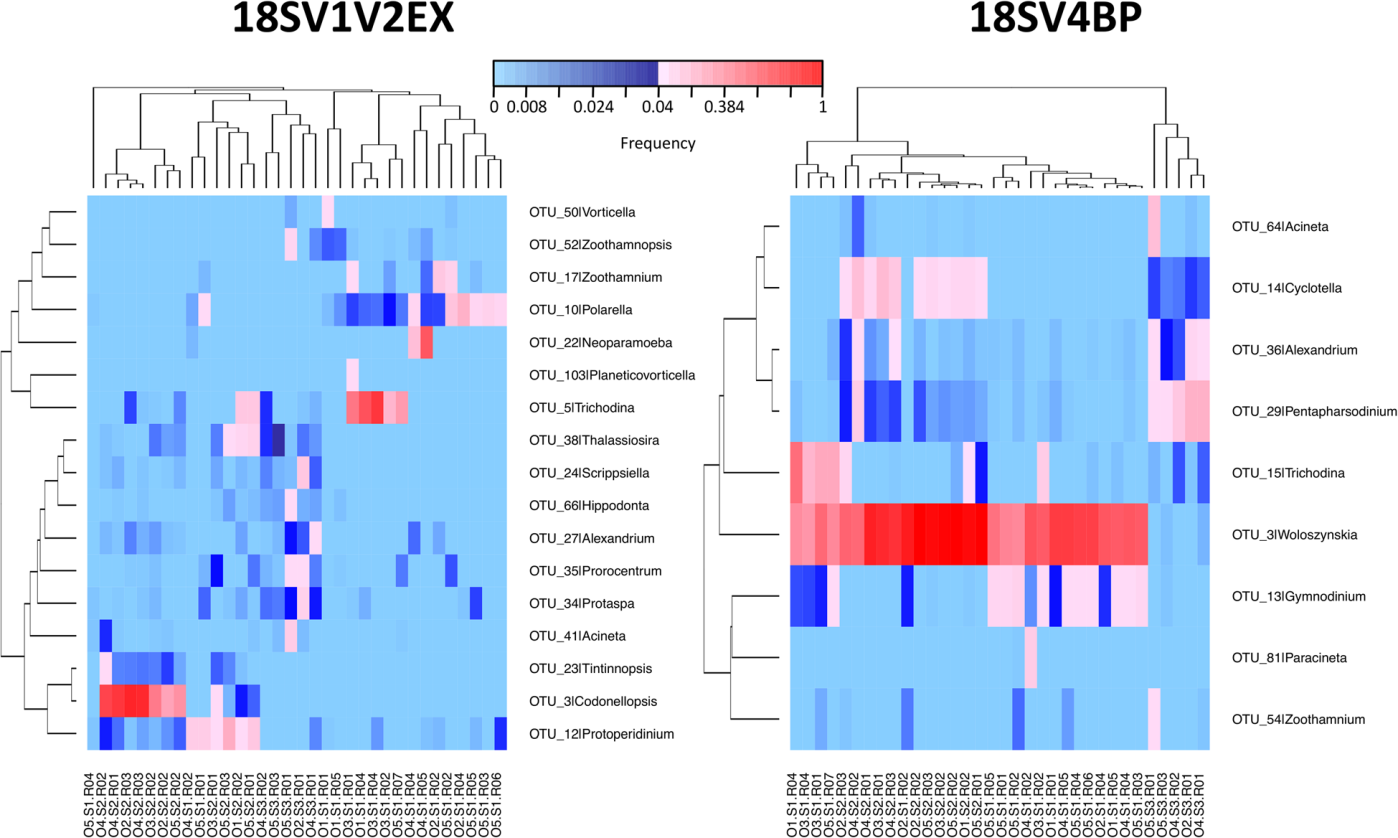
Heatmaps of dominant genera using 18SV1V2EX and 18SV4BP. Clustering were computed using the average linkage method. Bray-Curtis dissimilarities based on abundances of genera were used for samples. Distances based on Spearman’s *rho* correlation were used for protist genera. Only genera with a frequency above 4% in at least one sample are shown. Frequencies above and below 4% are displayed in red and blue, respectively

**Fig. 8:**
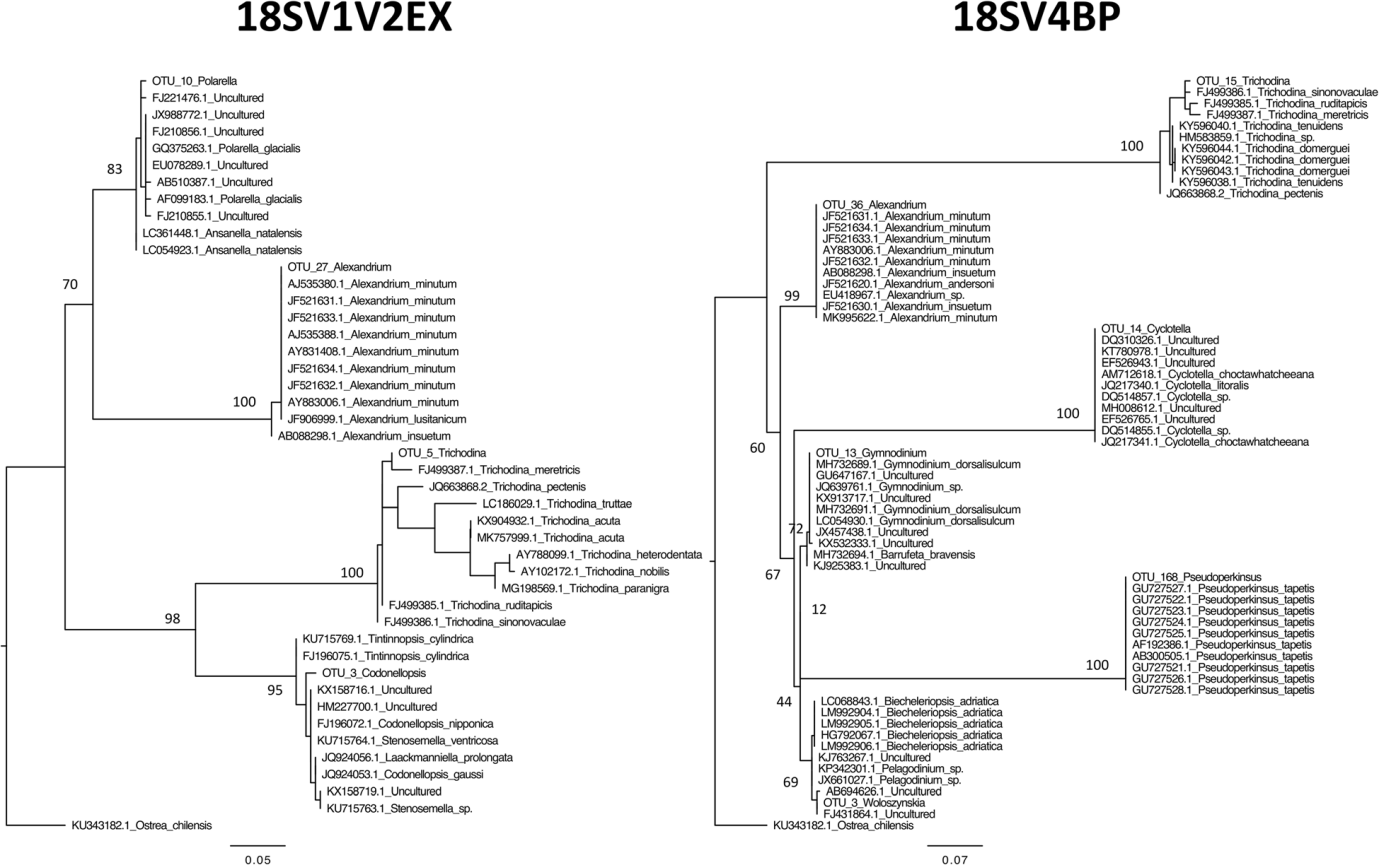
Maximum-likelihood phylogenetic trees of dominant genera. Trees were rooted using *Ostrea chilensis*. Numbers are ultrafast bootstraps (%) reflecting clade support of the main nodes

We computed heatmaps for both genetic markers to describe the distribution of dominant genera within oyster samples (Fig. 7). In addition to *Codonellopsis* and *Woloszynskia*, the main genera were *Cyclotella*, *Gymnodinium*, *Polarella*, and *Trichodina*. Notably, the potential pathogen *Alexandrium* was also found in high abundances within some samples (Fig. 7), and we also identified the potential pathogen *Pseudoperkinsus* at much lower abundances (Additional file 6: Table S4). Then, we compared the nucleotide sequences of these dominant genera to the nucleotide collection of NCBI using BLASTn, and we computed phylogenetic reconstructions using the first hits (Fig. 8). These analyses revealed the phylogenetic diversity of protists within oysters, and particularly that *Pseudoperkinsus* and *Trichodina* genera were similar to isolates already identified within different bivalve species (*Adipiocola pacifica*, *Crassostrea gigas*, *Mizuhopecten yessoensis*, *Mytilus* sp., and *Venerupis philippinarum*) (Table 3).

**Table 3:** Host annotations for BLASTn searches of dominant protist genera (NCBI)

Accession number	Description	Host	Marker
JX661027.1	*Pelagodinium* sp.	*Acanthochiasma* sp. (Radiolaria)	18SV4BP
AB300505.1	*Pseudoperkinsus tapetis*	*Adipiocola pacifica* (Mussel)	
GU727525.1		*Crassostrea gigas* (Oyster)	
GU727528.1			
GU727524.1		*Katharina tunicata* (Chiton)	
GU727527.1		*Leptosynapta clarki* (Sea cucumber)	
GU727522.1		*Mytilus* sp. (Mussel)	
GU727523.1			
GU727526.1		*Phascolosoma agassizii* (Peanut worm)	
GU727521.1		*Venerupis philippinarum* (Clam)	
KY596042.1	*Trichodina domerguei*	*Gasterosteus aculeatus* (Teleostei)	
KY596043.1			
KY596044.1			
KY596038.1	*Trichodina tenuidens*		
KY596040.1			
JQ663868.1	*Trichodina pectenis*	*Mizuhopecten yessoensis* (Bivalvia)	18SV4BP/ 18SV1V2EX
HM583859.1	*Trichodina* sp.	*Salmo salar* (Teleostei)	18SV4BP

## Discussion

### Advantages and disadvantages of blocking and excluding primers

The UNonMet primers were previously designed to exclude most metazoan sequences [16]. Our in silico analyses validated the estimated high performances of this primer set [17]. UNonMet primers had potentially two main limitations (Table 4). First, expected amplicon size was not compatible with current sequencing technology for metabarcoding. Secondly, while in silico analyses suggested that this primer set performed well, it was proposed that excluding primers might preclude as well the amplification of unexpected taxa, in particular from taxa not described so far [18].

**Table 4:** Advantages and disadvantages between blocking and excluding primers to study protists within oyster microbiota

	18SV4BP	UNonMet	18SV1V2EX
Advantages	BP specifically targets host sequences	No host sequences are expected to be amplified	No host sequences were amplified
		Protist diversity is expected to be well represented	High nucleotide polymorphism compared to 18SV4BP
Disadvantages	Host sequences were not completely removed	Excluding primers might not only exclude metazoans, but also other unexpected taxa	
		Expected amplicon size is too large for current Illumina MiSeq technology	
	Low nucleotide polymorphism compared to 18SV1V2EX		

Blocking primers are highly specific and prevent the amplification of a small range of sequences [18]. As a consequence, we decided to design blocking primer (18SV4BP) to study oyster microbiota, and to compare it in vivo (i.e., through metabarcoding analyses) to a primer set that was expected to exclude *C. gigas* sequences (18SV1V2EX) [20]. In silico analyses highlighted that the blocking primer was expected to prevent amplification of most Ostreoida and *Crassostrea* DNA. However, various efficiencies were observed for the different samples when we performed PCR. On average, oyster sequences still represented 83% (from 56 to 99%). Such variations were already observed in previous studies based on blocking primers [18–20], and they might be mainly related to the ratio between host and total DNA. In contrast, most host sequences were not amplified when using the excluding 18SV1V2EX primer set (< 1%). However, according to in silico analyses, we found that many non-metazoans (e.g., Archaeplastida, Excavata, and Picozoa) might not be amplified in comparison to 18SV4BP.

Altogether, these analyses revealed advantages and disadvantages for the three primer sets (Table 4). While blocking primers are less expected to exclude other sequences than the targeted ones [18], they did not remove all host DNA. In contrast, while excluding primers might possibly exclude unexpected taxa, the metabarcoding diversity was similar to blocking primers.

### Protist assemblages within oyster microbiota

Both blocking (18SV4BP) and excluding (18SV1V2EX) primers showed similar patterns of protist diversity using metabarcoding sequencing. No significant differences were found for alpha diversity indices, and protist compositions were significantly correlated between both primer sets at each taxonomic rank (from OTU to phylum). The major difference between protist assemblages was found at the genus level. In particular, *Codonellopsis* was dominant for 18SV1V2EX, but absent from 18SV4BP dataset. Surprisingly, this genus was already identified in *C. gigas* using morphology [29]. Because both 18SV1V2EX and 18SV4 are degenerated primer sets (Table 1), they might amplify differently some protist groups. Differences between marker regions were already observed in previous metabarcoding studies. For example, different protist assemblages were obtained between 18SV4 and 18SV9 marker regions when they were used to study coastal phytoplankton [22].

Alveolata, Stramenopiles and Archaeplastida were the main phyla within oyster microbiota. Other studies highlighted that these groups were highly abundant in seawater [30, 31]. Thus, it was difficult to conclude if these phyla were resident within oyster microbiota, or represent only environmental protists obtained through filtration activity [32]. However, potential oyster pathogens were identified in this study, such as the *Alexandrium* and *Pseudoperkinsus* genera. In addition, another dominant genus was already found to affect oysters, such as *Gymnodinium* that might cause oyster tissue injuries [33]. Lastly, BLASTn searches revealed that the *Trichodina* genus was similar to an isolate already identified within another bivalve species (*Mizuhopecten yessoensis*). Overall, these results highlighted the potential of these primer sets to study the whole eukaryotic microbes within oyster microbiota, but also diseases caused by protists.

## Conclusions

To conclude, we developed a blocking primer to study eukaryotic microbes within oyster microbiota, and we compared it to excluding primers. We found that the three primer sets had advantages and disadvantages, but they offered the possibility of targeting a compartment that was rarely described so far in most known microbiota. As a consequence, these primers are promising tools to better understand oyster homeostasis and disease development, such as the Pacific Oyster Mortality Syndrome (POMS) targeting juveniles.

## Methods

### Blocking and excluding primers

Blocking primers were designed for 18SV4 primer sets (Table 1) in order to reduce the proportion of host sequences. First, we downloaded the non-redundant (99%) Silva SSU database (release 128, September 2016) [34, 35]. Then we only kept sequences that matched with 18SV4 primer set (we allowed one mismatch because known sequences of *Crassostrea* differed from one position with this primer set). Based on annotations, sequences were divided into two databases: metazoan and non-metazoan. In order to design blocking primers that overlap the reverse primer and the 3′-region of host amplicons, we aligned the last 40 nucleotides (corresponding to the 3′-region of amplicon and the reverse primer) of *Crassostrea* sequences with the non-metazoan database using MAFFT (default parameters) [36]. According to previous studies, we designed several blocking primers having less than 30 bp, with 10 bp overlapping the reverse primer, and having a Tm similar to the targeted primer set [18, 19]. The best candidate was finally identified using specificity tests against metazoan and non-metazoan databases (i.e., targeting only oysters and no non-metazoans). Lastly, this primer was synthesized and modified at the 3′-end with a Spacer C3 CPG (3 hydrocarbons) [18].

In addition, we compared the specificity of 18SV4BP to two already published excluding primer sets: the UNonMet primers [16], and a primer set that targeted the variable loops V1 and V2 of the 18S rRNA gene [20]. To estimate the specificity of the three primer sets, we used the non-redundant (99%) Silva SSU database (release 128, September 2016). First, we randomly selected a sequence that matched with the three primer sets, and we used it as a query to identify sequences that matched with each amplicon in the Silva SSU database using BLASTn [37] (evalue< 10^− 5^). Secondly, BLASTn subjects were then aligned with the query sequence using MAFFT (default parameters) [36] to find sequences having the complete amplicon regions. Thirdly, we compared sequence annotations (metazoa and non-metazoa) between sequences that matched or not with the different primer sets.

### Biological material

Five biparental oyster families (hereafter named O1-O5) of *Crassostrea gigas* were used in this study (Additional file 1: Table S1). They were produced at the hatchery (Argenton, France) using a methodology that allowed the production of pathogen-free juveniles (please see reference [4] for more details). Individuals of each oyster family were either kept at the hatchery (please see reference [38] for more details) (sampling #1, Additional file 1: Table S1), or placed in the natural environment (Atlantic Ocean, latitude: 48.335263; longitude: 4.317922) for 5 days in April 2016 (sampling #2) or in July 2016 (sampling #3). Oysters were sampled, flash frozen in liquid nitrogen, and stored at − 80 °C.

### DNA extraction, PCR and sequencing

DNA extractions from frozen oysters were done using the DNA from tissue Macherey-Nagel kit (ref. 740,952.250) according to the manufacturer’s protocol (please see reference [38] for more details).

The rRNA genes were amplified and sequenced using the 18S variable V1V2 and V4 loops for eukaryotic communities (Table 1) [22, 39]. PCR reactions were carried in a 25 μl volume with final concentrations of 0.4 μM of each PCR primers, 0.02 U of the Qiagen Hotstar Taq DNA Polymerase, 0.2 mM of the dNTP mix and 1xTaq buffer. In order to reduce amplification of *C. gigas* amplicons for 18SV4, the blocking primer was added to the PCR mix at a final concentration of 1.2 μM (Table 1). PCR cycling included an initial incubation of 15 min at 96 °C followed by 35 cycles of 96 °C for 30 s, 52 °C for 30 s and 72 °C for 1 min, with a final 10 min incubation at 72 °C. Paired-end sequencing (250 bp) was done at the McGill University (Génome Québec Innovation Centre, Montréal, Canada) on the MiSeq system (Illumina, v2 chemistry) according to the manufacturer’s protocol. Raw sequence data are available in the SRA database (accession number PRJNA579900).

### Sequence analyses

We used the FROGS pipeline (Find Rapidly OTU with Galaxy Solution) implemented into a galaxy instance (http://sigenae-workbench.toulouse.inra.fr/galaxy/) to define Operational Taxonomic Units (OTUs), and compute taxonomic annotations [40] (please see reference [38] for more details). We filtered the dataset for singletons and we annotated OTUs using Blast+ against the Protist Ribosomal Reference database (PR^2^) [41]. Rarefaction curves of species richness were computed using the {phyloseq} R package and the ggrare function. The rarefy_even_depth function was used to subsample dataset to 5160 reads per sample using. We did not compare low coverage samples (< 5160 sequences). The estimate_richness function was used to compute alpha diversity metrics (Observed, Chao1 and Shannon). Pielou’s measure of species evenness was obtained using the diversity function {vegan}. In order to compare length and nucleotide diversity of 18SV1V2EX and 18SV4BP amplicons, protist sequences from subsampled dataset were aligned for each region using MAFFT, and alignments were trimmed at each extremity. Then, nucleotide diversity of OTU sequences was computed using the nuc.div function {pegas}. The tax_glom function was used to obtain abundances at differents taxonomic ranks (from genus to phylum). Multi-affiliations were not considered for these taxonomic ranks. Then, Bray-Curtis dissimilarities were computed at each taxonomic rank to study differences between samples for protist compositions (beta diversity) (vegdist function, {vegan}).

### Phylogenetic reconstructions

We performed BLASTn searches of the dominant protist genera against the non-redundant nucleotide collection of NCBI. We kept the 10 first hits for each query (coverage and identity > 90%) to compute phylogenetic reconstructions. Sequences were aligned using MAFFT [36], and trimmed at each extremity. Poorly aligned and highly variable regions of the alignment were automatically removed using GBlocks [42], and maximum likelihood (ML) trees were computed with IQ-TREE v1.3.8 using the best model (selected with the Bayesian information criterion) [43], and validated via a ultrafast bootstrap procedure with 1000 replicates [44].

### Statistical analyses

All statistical analyses were done using R v3.3.1 [45].

Hierarchical clusterings (average linkages (hclust {stats})) were computed to describe composition of microbial communities between samples using Bray-Curtis dissimilarities (vegdist {vegan}). Clusterings of 18SV1V2EX and 18SV4BP were plotted face to face using the tanglegram function {dentextend}. Heatmaps of dominant protist genera were computed using relative abundances and the heatmap.2 function ({gplots}).

We performed Student’s t-test (t.test {stats}) or non-parametric Wilcoxon test (wilcox.test {stats}) (when normality was rejected with the Shapiro-Wilk test, (shapiro.test {stats})) to compare (i) amplicon sizes, (ii) abundances of total sequences, (iii) abundances of high taxonomic ranks (oyster, Embryophyceae, protists, others), and (iii) alpha diversity metrics (Chao1, evenness and Shannon) between 18SV1V2EX and 18SV4BP. Mantel test (mantel {vegan}) was used to compare 18SV1V2EX and 18SV4BP dissimilarities (Bray-Curtis index) at each taxonomic rank. Permutational multivariate analysis of variance (PERMANOVA, adonis2 {vegan}) was used to investigate the variation of the different OTUs under the constraint of environmental conditions (S1-S3) and oyster families (O1-O5).

## Supplementary information

**Additional file 1: Table S1.** Microbiota samples

**Additional file 2: Figure S1.** Sequences of high taxonomic ranks (oysters, Embryophyceae, protists) within oyster sample

**Additional file 3: Table S2.** Number of sequences. Values correspond to 18SV1V2EX/18SV4BP.

**Additional file 4: Figure S2.** Rarefaction analyses.

**Additional file 5: Table S3.** OTU annotations and abundances for 18SV1V2EX samples.

**Additional file 6: Table S4.** OTU annotations and abundances for 18SV4BP samples.

**Additional file 7: Figure S3.** Comparison of amplicon sizes between 18SV1V2EX and 18SV4BP

**Additional file 8: Table S5.** Metadata and alpha diversity indices. Alpha diversity values correspond to 18SV1V2EX/18SV4BP. NA: not analysed (protist sequences< 5160).

## Data Availability

The sequences can be accessed at the Sequence Read Archive repository (https://www.ncbi.nlm.nih.gov/sra/) with BioProject ID PRJNA579900. Until then, the sequences are available from the corresponding author upon reasonable request.

## Abbreviations

18SV1V2: Variable loops V1 and V2 of the 18S rRNA gene
18SV1V2EX: Sequences obtained using the excluding primer set targeting 18SV1V2
18SV4: Variable loop V4 of the 18S rRNA gene
18SV4BP: Sequences obtained using the blocking primer targeting the 18SV4 primer set
OsHV-1: *Ostreid herpesvirus* 1
OTU: Operational taxonomic units
POMS: Pacific oyster mortality syndrome
PR^2^: Protist ribosomal reference database
SSU rRNA: Small subunit ribosomal ribonucleic acid
UNonMet: Universal non-metazoa excluding primers
