# Supplementary figures and images for: Unveiling protist diversity associated with the Pacific oyster Crassostrea gigas using blocking and excluding primers
Source: BMC Microbiol. 2020 Jul 3;20:193. doi: 10.1186/s12866-020-01860-1 (PMC7333408; doi:10.1186/s12866-020-01860-1)

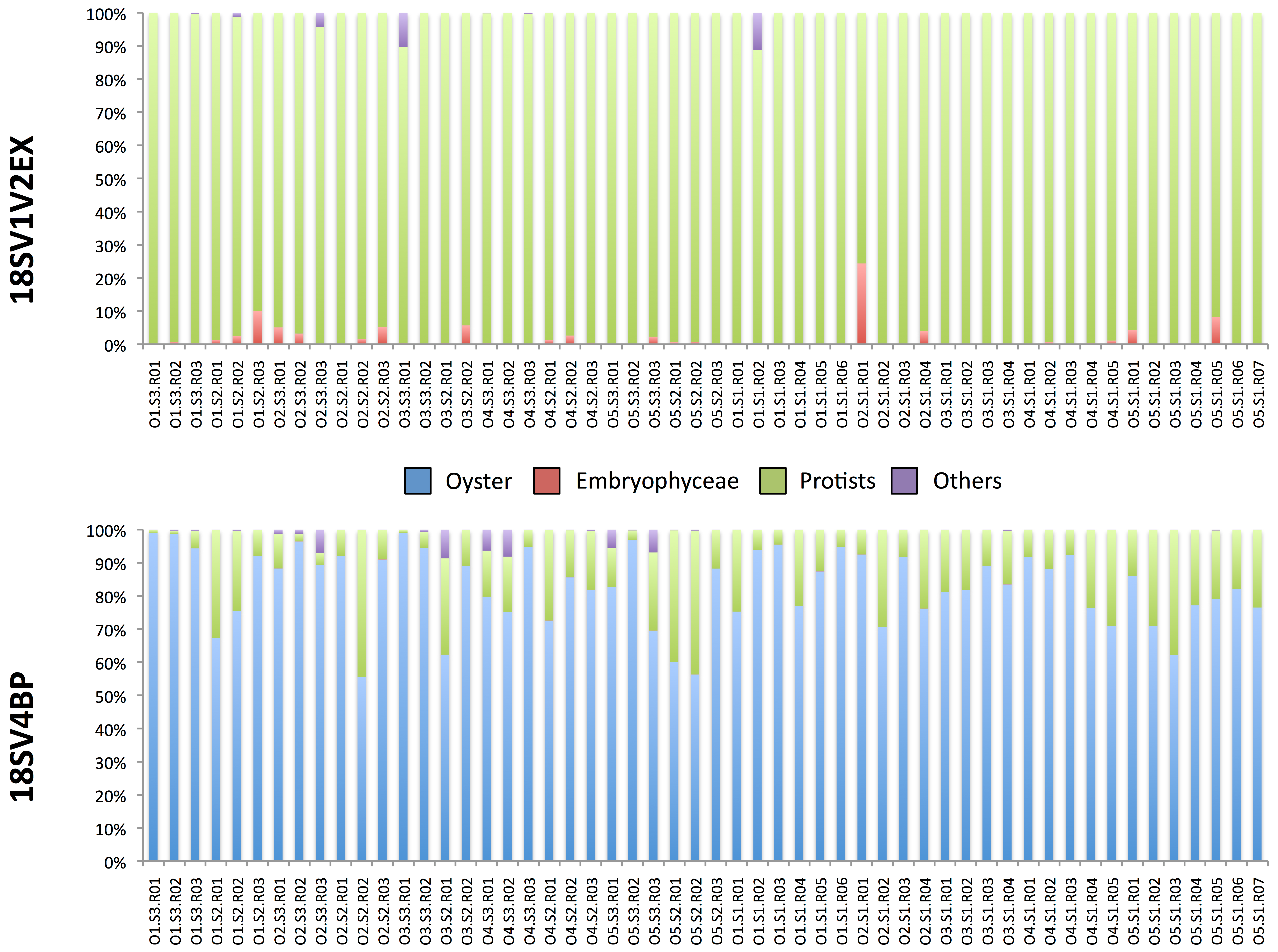

Supplement: Supplementary file 2 — Additional file 2: Figure S1. Sequences of high taxonomic ranks (oysters, Embryophyceae, protists) within oyster sample [file 12866_2020_1860_MOESM2_ESM.tiff]

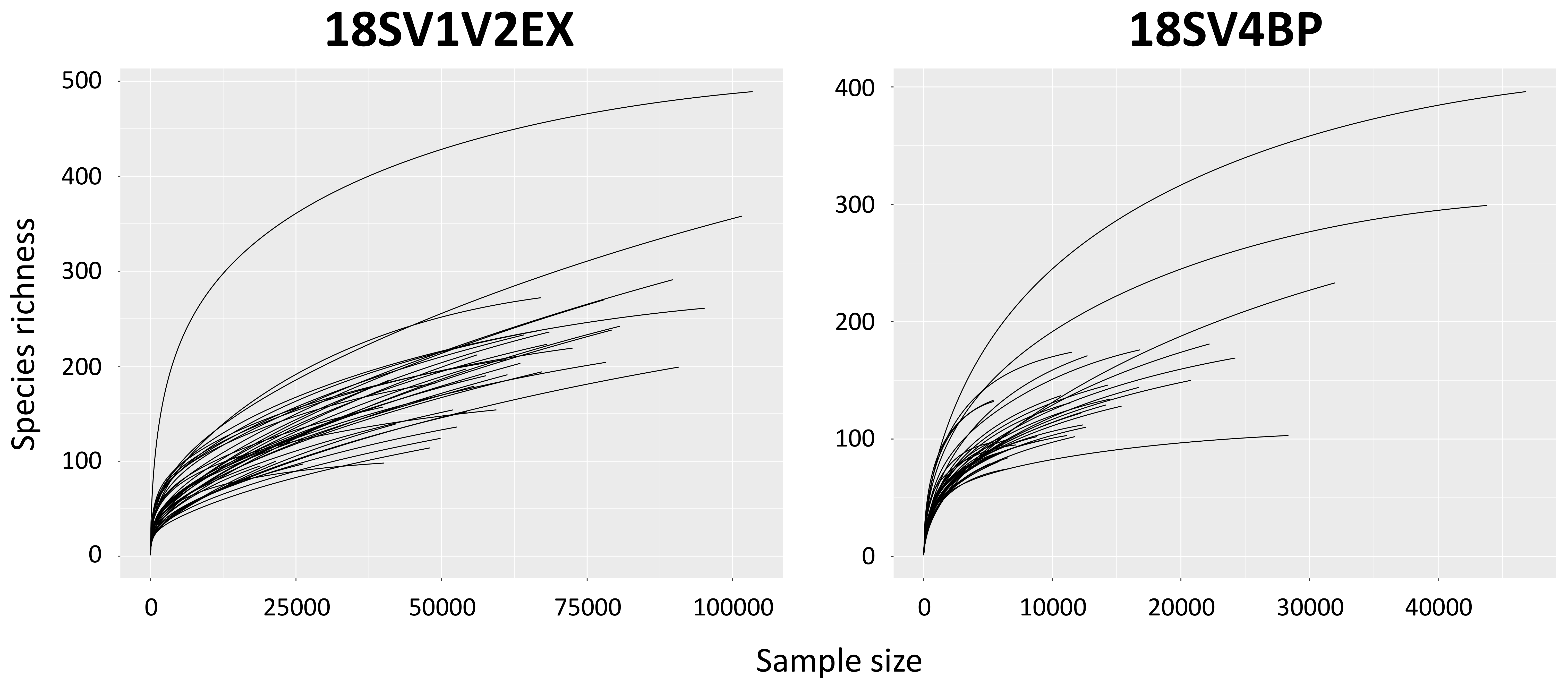

Supplement: Supplementary file 4 — Additional file 4: Figure S2. Rarefaction analyses. [file 12866_2020_1860_MOESM4_ESM.tiff]

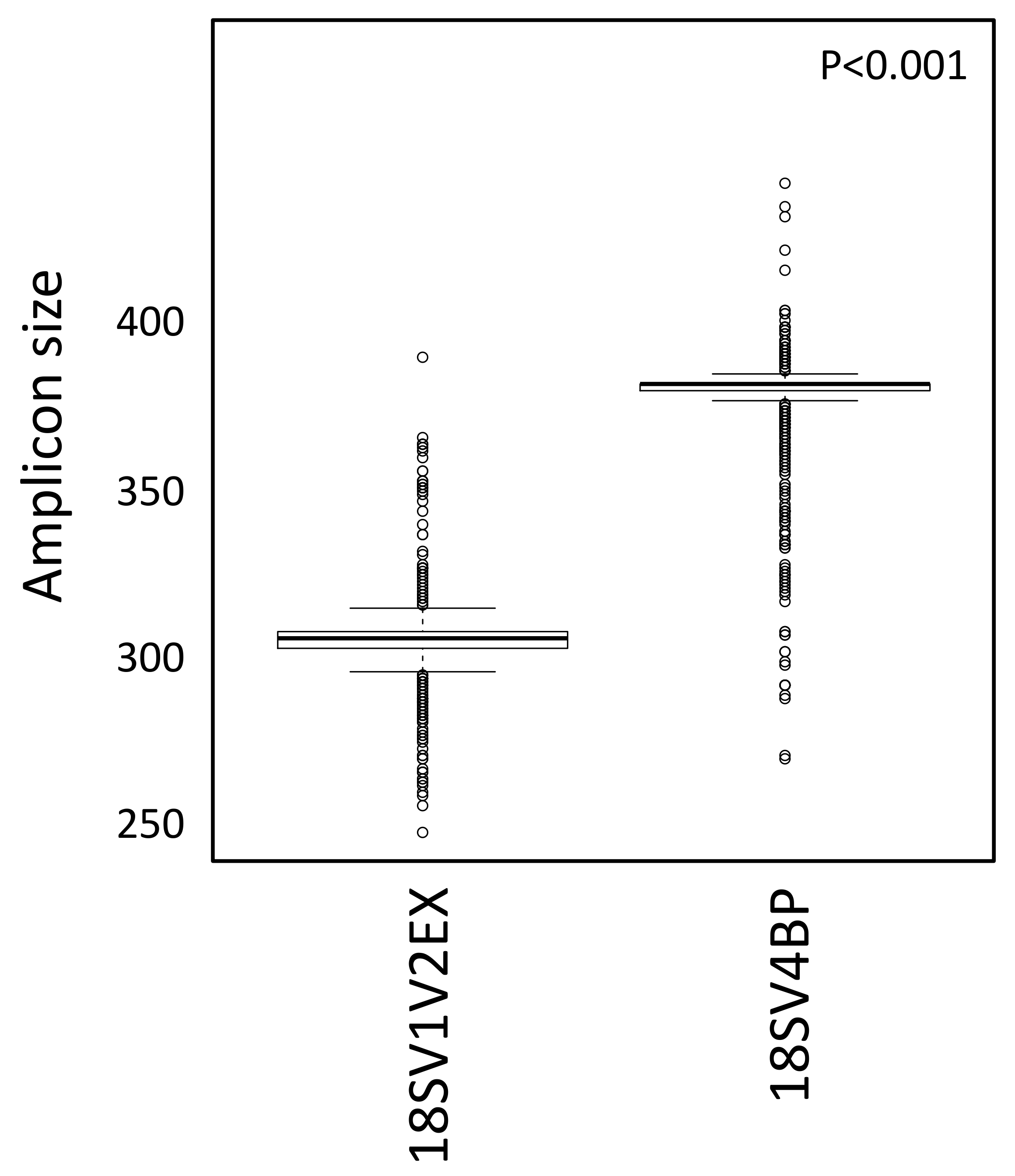

Supplement: Supplementary file 7 — Additional file 7: Figure S3. Comparison of amplicon sizes between 18SV1V2EX and 18SV4BP [file 12866_2020_1860_MOESM7_ESM.tiff]
